# 2682. Opt-Out Syphilis Screening in an Urgent Care Center Increases Recognition and Treatment of Asymptomatic Syphilis Infection and Uptake of HIV Pre-Exposure Prophylaxis

**DOI:** 10.1093/ofid/ofad500.2293

**Published:** 2023-11-27

**Authors:** Stephanie F Sweitzer, Joseph Sharp, Judah Gruen

**Affiliations:** University of North Carolina, Chapel Hill, North Carolina; Emory University School of Medicine, Greenville, Georgia; University of Texas Southwestern Medical School, Dallas, Texas

## Abstract

**Background:**

Atlanta, Georgia has an extremely high burden of both HIV and early syphilis. This syndemic is driven by a complex set of structural factors that result in striking healthcare inequities, further burdening communities with low access to primary care. These populations may instead utilize emergency departments and urgent care centers (UC) for medical care. UC visits represent a crucial opportunity for recognition and treatment of syphilis as well as connection to HIV prevention services. We describe a novel pilot of a universal, opt-out syphilis screening program offered to all adults in the Grady Urgent Care Center in Atlanta.

**Methods:**

A retrospective chart review was performed on all patients 18 years and older who had a rapid plasma reagin (RPR) test collected from 9/1/21 to 12/31/21. Demographic data, syphilis diagnosis, syphilis treatment, and HIV testing and serostatus were abstracted from the electronic health record. Patients with reactive RPRs were contacted by a physician for diagnosis and potential syphilis treatment, sexual health counseling, and referral for HIV pre-exposure prophylaxis (PrEP) or HIV care.

**Results:**

From 9/1/21 to 12/31/21, 5794 patients were triaged and 1381 underwent RPR screening (23.8%). Eighty (5.8%) had reactive RPRs, and 42 (52.5%) had active syphilis infection. Of those with active syphilis, 39 (92.9%) received treatment, and 35 (83.3%) completed treatment. Among all undergoing RPR screening, 37 (2.7%) were living with HIV. Of those who were not previously diagnosed with HIV, 1279 (93.9%) underwent concurrent HIV testing. Among all undergoing RPR screening, 41 expressed interest in PrEP, and 7 completed PrEP clinic intake. Of those with reactive RPRs, 16 expressed interest in PrEP, and 6 completed PrEP clinic intake.

**Figure:**
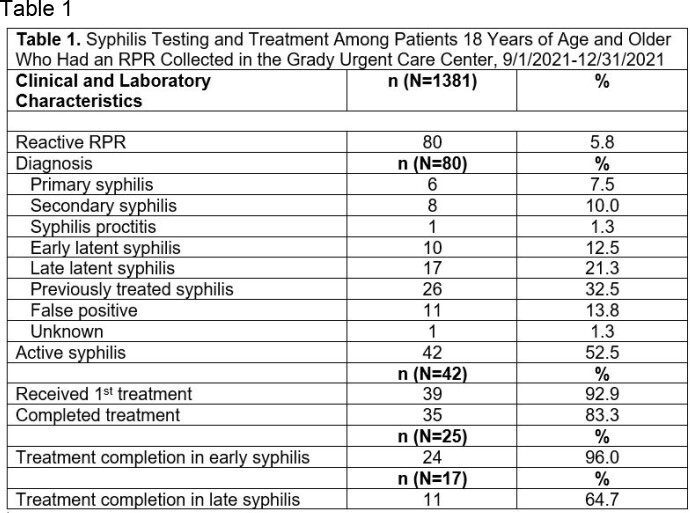

Syphilis Testing and Treatment Among Patients 18 Years of Age and Older Who Had an RPR Collected in the Grady Urgent Care Center, 9/1/2021-12/31/2021

**Figure:**
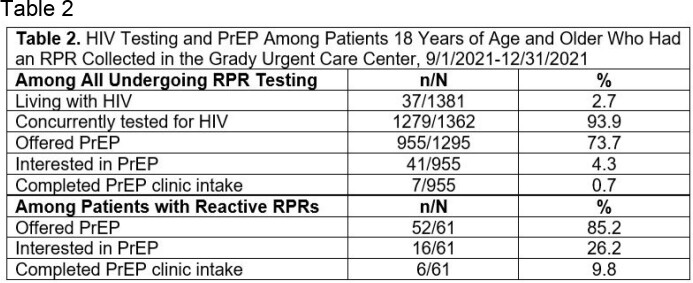

HIV Testing and PrEP Among Patients 18 Years of Age and Older Who Had an RPR Collected in the Grady Urgent Care Center, 9/1/2021-12/31/2021

**Conclusion:**

Routine, opt-out syphilis testing was used to identify a substantial number of patients with active syphilis and connect a majority to treatment, underscoring the need for universal syphilis screening in this setting. It also provided an opportunity to deliver sexual health counseling and offer PrEP, although few patients completed PrEP clinic intake, suggesting that a more targeted approach to PrEP referral may be more effective.

**Disclosures:**

**All Authors**: No reported disclosures

